# Prospective Evaluation of a Topical Botanical Skin Care Regimen on Mild to Moderate Facial and Truncal Acne and Mood

**DOI:** 10.3390/jcm12041484

**Published:** 2023-02-13

**Authors:** Yvonne Nong, Nimrit Gahoonia, Julianne Rizzo, Waqas Burney, Raja K. Sivamani, Jessica Maloh

**Affiliations:** 1 Integrative Skin Science and Research, Sacramento, CA 95815, USA; 2 College of Human Medicine, Michigan State University, East Lansing, MI 48824, USA; 3 College of Osteopathic Medicine, Touro University, Vallejo, CA 94592, USA; 4 School of Medicine, University of California Davis, Sacramento, CA 95817, USA; 5 Pacific Skin Institute, Sacramento, CA 95815, USA; 6 Department of Dermatology, University of California Davis, Davis, CA 95816, USA

**Keywords:** botanical, acne vulgaris, topical, regimen, skincare

## Abstract

Acne vulgaris is a common inflammatory condition that can be associated with profound psychosocial impacts. Conventional treatment includes topical retinoids, benzoyl peroxide, and antimicrobials, and some may cause irritation and skin dryness. In this 8-week open-label study, we examined the effects of a botanical skin care regimen (Codex Labs Shaant Balancing regimen) on mild to moderate facial and truncal acne. Twenty-four male and female subjects between the ages of 12 and 45 years were assessed for eligibility, 20 were enrolled, and 15 completed all study visits. Facial and truncal acne lesion counts, skin hydration, sebum excretion rate, and mood were assessed at baseline, week 4, and week 8. Total facial lesion counts (inflammatory and non-inflammatory lesions) decreased by 20.5% at week 4 (*p* = 0.06) and by 25.2% at week 8 (*p* < 0.05). Inflammatory lesion counts on the trunk were found to decrease at week 8 relative to baseline by 48% (*p* < 0.05). Forehead sebum excretion rate decreased by 40% at week 4 (*p* = 0.07) and 22% at week 8 (*p* = 0.08), and cheek skin hydration increased by 27.6% at week 4 (*p* = 0.14) and 65% at week 8 (*p* = 0.10). Participants also experienced significant improvement in components of a positive effect, such as feeling “strong” and “inspired”, and a decrease in negative effects, such as feeling “irritable.” Overall, the botanical skin care regimen was found to be well-tolerated. Our study suggests that a botanical skin care regimen may reduce facial and truncal acne lesion counts, increase skin hydration, reduce sebum production, and augment positive effects and moods in those with mild to moderate facial and truncal acne.

## 1. Introduction

Acne vulgaris is one of the most common dermatologic conditions, affecting over 85% of adolescents and young adults, and it can have major impacts on both physical and mental health [1]. The pathogenesis of acne is multifaceted; prime contributors include the colonization with *Cutibacterium acnes (C. acnes)*, overactive sebum production, follicular hyperkeratinization, and inflammation [2]. Acne is typically present on the face, shoulders, chest, and back with inflammatory lesions such as papules and pustules, along with non-inflammatory lesions such as open or closed comedones [3,4]. As many of these lesions heal, they can leave behind post-inflammatory hyperpigmentation and scarring [5]. Acne is associated with feelings of low self-esteem, decreased confidence, depression, and anxiety, with a quality of life that is impacted negatively overall [6,7].

Common first line treatment options for acne include topical retinoids and topical antimicrobial products [8]. One of the side effects of retinoids is skin irritation [9]. Topical antimicrobials include benzoyl peroxide, clindamycin, and erythromycin [10]. Antibiotics can lead to the development of drug-resistant bacteria [11,12], and the regular use of benzoyl peroxide to reduce the development of drug-resistant bacteria may cause drying and scaling, which can lead to discomfort and irritation [13]. Given these undesirable effects, combination therapy or alternative therapies for acne that include naturally derived ingredients have garnered public interest. While there is a sentiment that botanicals and naturally derived products may not be as efficacious, there is growing evidence that botanicals and naturally derived ingredients can be efficacious in the treatment of acne [14,15,16].

One of the primary drivers for the natural skin care market is the growing awareness about the benefits of botanically derived ingredients [17]. For example, *Centella asiatica* has been found to offer anti-inflammatory effects in acne, while also improving skin dryness and irritation [18]. Bakuchiol is a plant-derived phytochemical that was found to have comparable effects to topical retinoids in improving wrinkles and hyperpigmentation but with less facial irritation than retinoids [19]. Extracts from biotech amplified *Tetraselmis* algae have well-characterized antioxidant properties [20]. Additionally, natural ingredients with absorbent properties such as bentonite may be helpful for managing excess sebum production and have demonstrated anti-inflammatory and skin regenerative effects [21]. Based on these findings, we investigated the impact of a botanical-based regimen that contained *Centella asiatica, Tetraselmis chui*, and bakuchiol on its effect on mild-to-moderate acne and its influence on mood.

## 2. Methods

### 2.1. Subjects

This study was conducted between March 2022 and December 2022 as an 8-week open-label study. Institutional Review Board approval was received on 5 March 2022 by Allendale, and the study was listed on clinicaltrials.gov (NCT05271487). Written informed consent was received from all participants prior to enrollment. Subjects from the greater Sacramento region were recruited. Inclusion criteria included males and females between the ages of 12 and 45 years old, with mild-to-moderate acne classified by an investigator global assessment (IGA) of 2 or 3, along with the presence of at least 10 inflammatory lesions and at least 15 total acne lesions. Subjects who had more than two nodules were excluded. Those with severe acne (IGA ≥ 4), women who were pregnant or breastfeeding, those who were current smokers or had a smoking history of >10 pack-years, those unwilling to discontinue facial products except for what is provided in the study, and those who changed their hormonal-based contraception within 3 months prior to enrollment were excluded from the study. Those who had isotretinoin use within the 3 months prior to joining the study and those who were unable to discontinue oral antibiotic, probiotic, topical antibiotics, and topical benzoyl peroxide use were also excluded from the study. Patients were advised to not seek any cosmetic treatments during the study or other medicated acne products.

### 2.2. Investigational Products

The skin care regimen consisted of a cleanser, oil control cream, exfoliator, toner, spot treatment, clay mask, and body scrub, commercially available as the Shaant collection (Codex Labs, Menlo Park, CA, USA). Compliance was assessed using a product log where participants were asked to make note of each time they used a product on a given day. Participant instructions for product use are outlined in Table 1.

### 2.3. Study Visits and Procedures

Written consent and assent were obtained prior to enrollment. Subjects were asked to undergo a 2-week washout from topical antibiotics or benzoyl peroxide use or a 4-week washout for oral probiotic supplements or oral antibiotic use. The study consisted of a total of 4 visits (a screening visit, a baseline visit, a visit after 4 weeks of product use, and a visit after 8 weeks of product use).

At baseline, week 4, and week 8, facial and truncal lesion count for inflammatory and non-inflammatory lesions was performed by a trained doctor or board-certified dermatologist. During these visits, biophysical features such as stratum corneum hydration and sebum excretion rate were obtained using the MoisturemeterSC^®^ (Delfin Technologies, Kuopio, Finland) and the Sebumeter^®^ (Courage+Khazaka electronic GmbH, Köln, Germany), respectively. Facial photographs were also captured at baseline, week 4, and week 8 using BTBP 3D Clarity Pro^®^ Facial Modeling and Analysis System (Brigh-Tex BioPhotonics, San Jose, CA, USA).

Mood was assessed at baseline, week 4, and week 8 using the validated Positive and Negative Affect Schedule (PANAS) questionnaire. This 20-item survey asked respondents to rate how often they felt each adjective in the prior week using the following 5-point Likert-type scale: 1 = “very slightly or not at all”, 2 = “a little”, 3 = “moderately”, 4 = “quite a bit”, and 5 = “extremely”.

At week 4 and week 8 visits, tolerability was evaluated using an 11-question survey inquiring about potential symptoms (i.e., itching, burning, stinging, scaling, redness, hypo- or hyperpigmentation) experienced with the product use. Participants were asked to rate these symptoms on a 3-point scale, with “0” indicating none, “1” as mild, “2” as moderate, and “3” as severe.

## 3. Results

**Figure 1:**
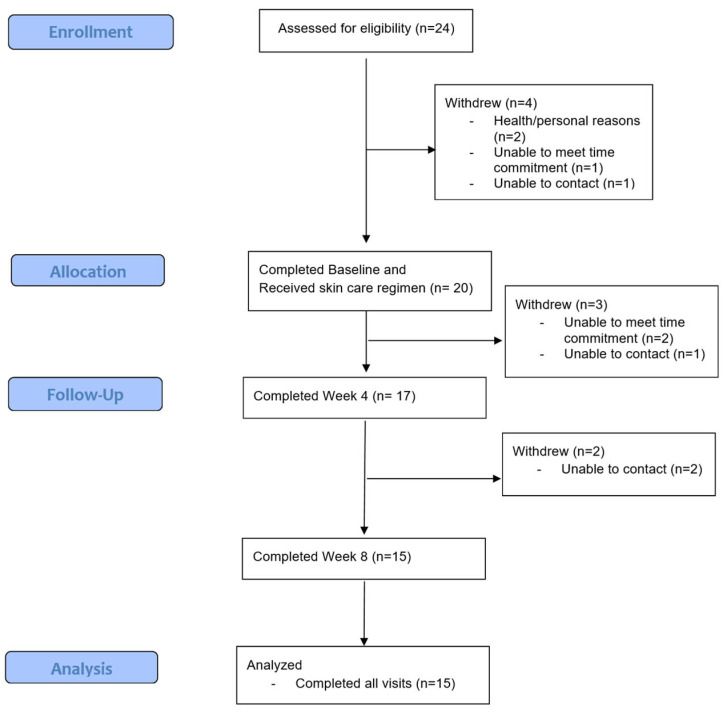
CONSORT (Consolidated Standards of Reporting Trials) flow diagram.

Out of 24 eligible participants, 4 did not enroll after screening, and 20 enrolled into the study. A total of 15 completed all visits per-protocol, 2 withdrew due to inability to meet time commitment, and 3 were lost to follow up (Figure 1). The majority of participants were female (23/24), and the mean age was 24.4 ± 7.3 years. The mean IGA severity at enrollment was 2.7.

### 3.1. Facial and Trunk Lesion Counts

**Figure 2:**
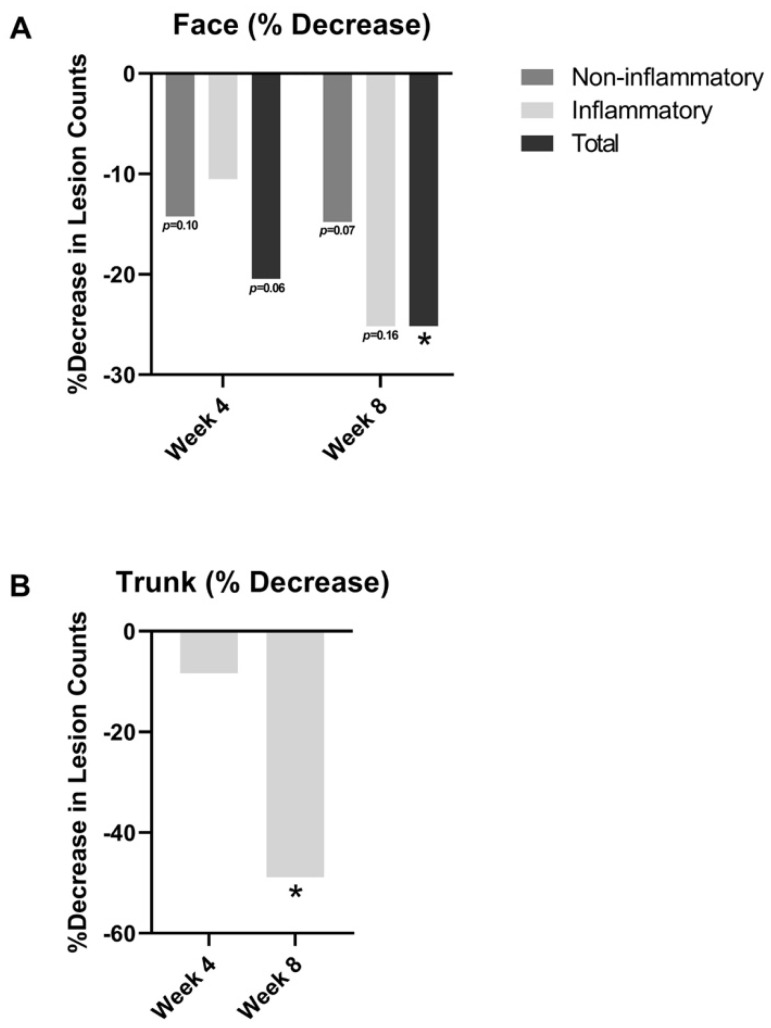
(**A**) Facial acne lesion counts and truncal lesion counts; (**B**) truncal acne inflammatory lesion counts at week 4 and week 8 relative to baseline. * = *p* < 0.05.

Lesion counts improved from baseline at both week 4 and week 8 in Figure 2. For facial acne (Figure 2A), the number of non-inflammatory lesions decreased by 14.2% at week 4 (*p* = 0.10) and by 14.8% at week 8 (*p* = 0.07) relative to baseline. Inflammatory lesions decreased by 10.5% at week 4 (*p* = 0.44) and by 25.2% at week 8 (*p* = 0.16). Total lesion counts (inflammatory and non-inflammatory lesions) decreased by 20.5% at week 4 (*p* = 0.06) and significantly by 25.2% at week 8 (*p* < 0.05) relative to baseline.

With regards to truncal acne (Figure 2B), there was also an 8.4% reduction in inflammatory lesions at week 4 relative to baseline (*p =* 0.39) and a 48.9% reduction at week 8 (*p* < 0.05) relative to baseline. 

**Figure 3:**
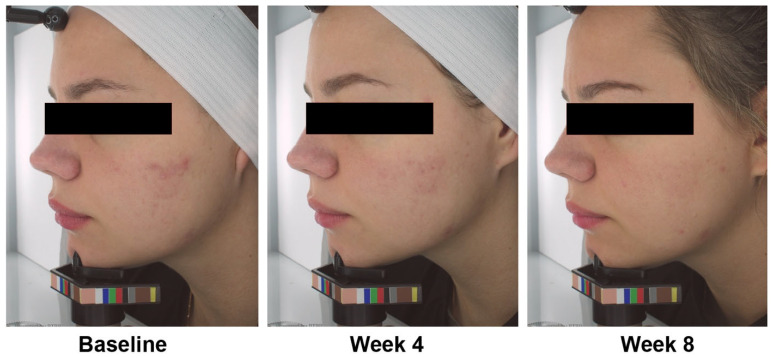
Acne photographs from a representative participant at baseline, week 4, and week 8.

Representative photo of the results is shown in Figure 3. 

### 3.2. Sebum Excretion Rate

**Figure 4:**
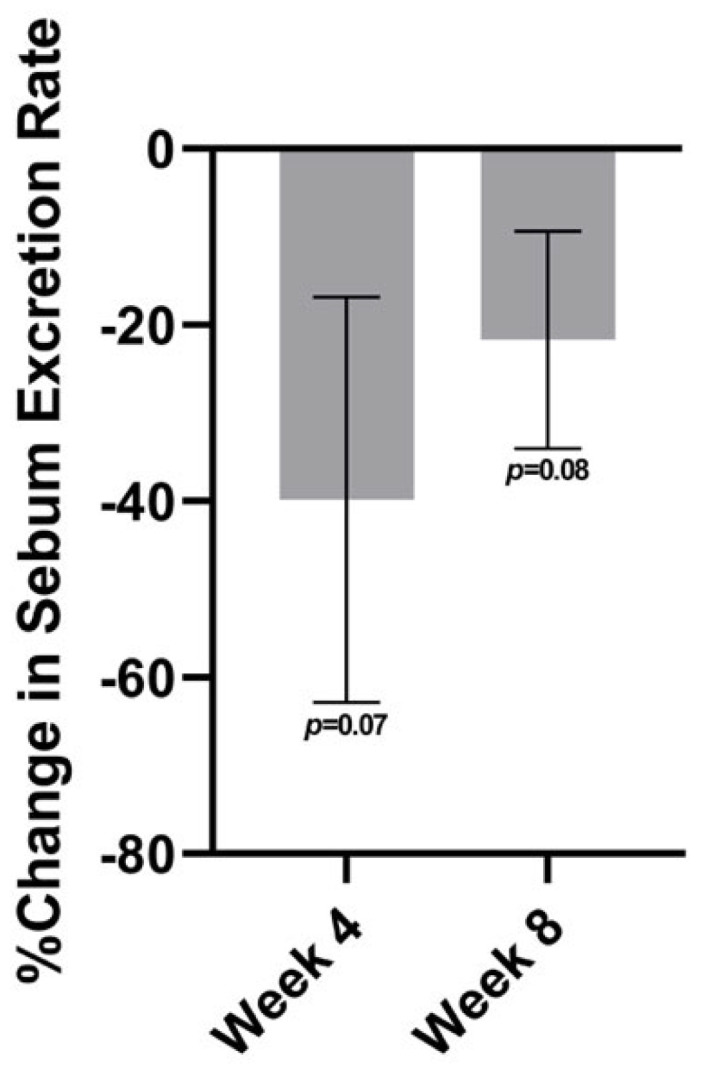
The sebum excretion rate at week 4 and at week 8 relative to baseline on the forehead.

The sebum excretion rate was found to have a decreasing trend on the forehead at both follow-up visits relative to baseline (Figure 4). From baseline to week 4, the average percent change was nearly a 40% reduction on the forehead (*p =* 0.07) and nearly a 35% reduction on the cheeks (*p =* 0.08). At week 8, there was a decrease in sebum excretion of about 22% on the forehead (*p* = 0.08).

### 3.3. Skin Hydration

**Figure 5:**
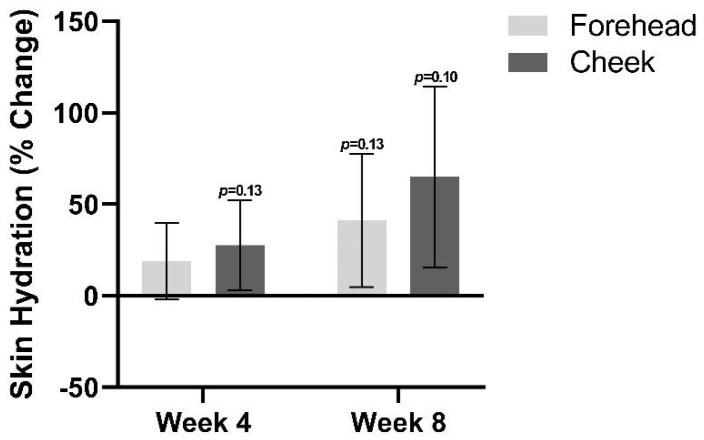
Skin hydration at week 4 and week 8 relative to baseline.

Skin hydration trended towards an increase at both follow-up visits relative to baseline (Figure 5). By week 4, there was an average increase in skin hydration of 19.1% on the forehead (*p* = 0.19) and an increase of 27.6% on the cheeks (*p* = 0.14). At week 8, a 41.1% increase in skin hydration was found on the forehead (*p* = 0.14), and a 65% increase was found on the cheek (*p* = 0.10).

### 3.4. Mood and Affect Score

**Figure 6:**
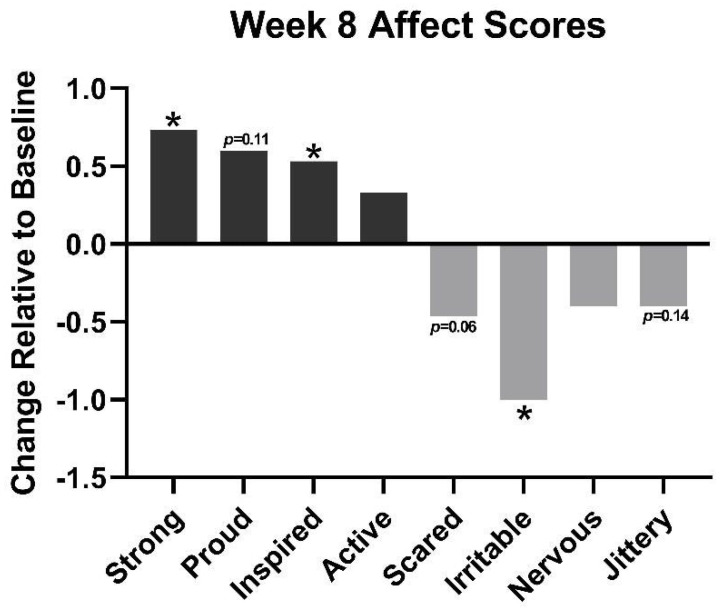
Components of positive effects (strong, proud, inspired, active) and negative effects (scared, irritable, nervous, jittery) at week 8 relative to baseline. * = *p* < 0.05.

After 8 weeks of use, the acne regimen was found to improve components of positive and negative effects (Figure 6). Overall, participants had an average 0.73 point increase in feeling “strong” (*p* ≤ 0.05) and a 0.53 increase in feeling “inspired” (*p* < 0.05). Additionally, at week 8, negative effects decreased, such as feeling “scared” was found to decrease by 0.46 (*p* = 0.06), while feeling “irritable” decreased by 1 point (*p* < 0.05).

### 3.5. Tolerability

**Figure 7:**
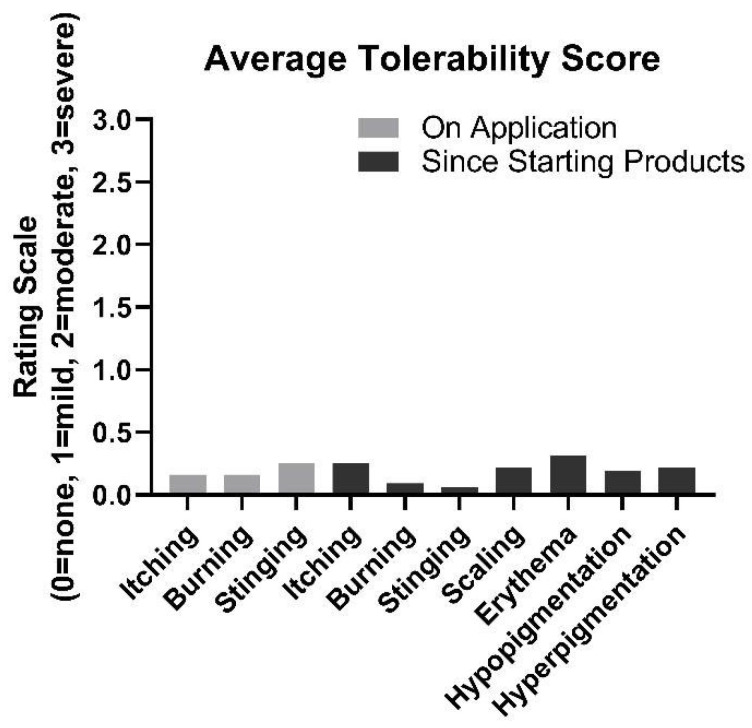
Average tolerability ratings from participants at week 8.

Overall, the products were found to be well-tolerated (Figure 7). On average, after 8 weeks of product use, participants rated symptoms of itching, burning, and stinging at 0.16, 0.16, and 0.25, respectively, on a 3.0 scale where 0 = no symptoms and 3 = severe. Scaling, erythema, hypopigmentation, and hyperpigmentation were almost never observed, on average, while using the product. No adverse effects were reported.

## 4. Discussion

There is a growing interest in natural, plant-based products for acne, yet few studies have sought to understand the effects of botanical compounds to help prevent and treat this common skin condition. Most clinical trials have focused on individual ingredients rather than a formulation or skincare regimen. This study examined the effect of a combination of botanical products intended to target various components of acne pathophysiology such as inflammation, *C. acnes* colonization, oil production, and follicular hyperkeratinization.

In this open-label clinical trial, a botanical-based skin care regimen improved the lesion count in those with mild-to-moderate acne. Both non-inflammatory and inflammation improved with this regimen, showing that both the comedones and papules of acne may improve with treatment. Moreover, there was a trend towards increased skin hydration and a trend towards decreased sebum excretion rate, which suggests that these products may help to control acne for oily skin types without drying the skin. This trend contrasts that of benzoyl peroxide, which generates free radicals in the skin and may damage the cutaneous barrier [22]. A study found that benzoyl peroxide-induced oxidative stress was attenuated when mice were pre-treated with spearmint extracted from the *Mentha spicata* plant [23]. This suggests that plant-based ingredients, especially those with antioxidant and anti-inflammatory properties, may help to counteract unwanted side effects associated with mainstay treatment options for acne. Although benzoyl peroxide was not tested in this particular regimen, it would be interesting to see if this regimen may allow for benzoyl peroxide to be used along with this regimen.

Many plant-based ingredients have been shown to synergistically target multiple pathways contributing to acne development and help explain the improvement in both the inflammatory and non-inflammatory lesions noted in this study. Tannins, such as those found in *Hamamelis virginiana* (or witch hazel), which is in the study toner product, act as an astringent with anti-inflammatory properties and have been shown to inhibit *C. acnes*-induced inflammation [24]. In addition, bakuchiol, derived from the leaves and seeds of the *Psoralea corylifolia* plant, is found in the balancing oil control cream and targets the inflammatory pathways in acne while also reducing skin discoloration [25]. For example, one study found that a cream containing 0.5% bakuchiol reduced the number of inflammatory lesions while improving existing post-inflammatory hyperpigmentation in a cohort of subjects with Fitzpatrick skin types III-VI with mild-to-moderate acne [25]. Because bakuchiol may have retinoid-like properties [26], it may target follicular hyperkeratinization as well. Bakuchiol is typically well tolerated at doses of 1%, as used in the botanical regimen studied here. The botanical regimen also utilizes *Tetraselmis chui*, which is an algae that has been reported to reduce sebum production on the face [27].

Another important aspect of the study regimen and study results worth highlighting is the Shaant Balancing Body Scrub, which was applied to the back, chest, and shoulders. Studies suggest that, while approximately 50% of facial acne patients also experience truncal acne, the trunk region tends to be overlooked in clinical trials [28,29]. The standard treatment option for truncal acne has been topical benzoyl peroxide, but there may be concern around the risk of bleaching clothes and the tendency toward skin irritation [29,30]. The results here suggest another option for those that are seeking treatment for truncal acne without an oral or systemic treatment. 

With regards to the spot treatment, one of the main ingredients is sulfur. This can sometimes be found in combination with sodium sulfacetamide or may also be used alone. Mainly used in formulations for its anti-inflammatory benefits, research has shown that a sodium sulfacetamide/sulfur emollient foam can markedly reduce the *C. acnes* colony count in vitro [31]. 

Additionally, an ingredient commonly found in all of the products is patchouli extract from the *Pogostemon cablin* plant [32]. Aside from its pleasant smell, patchouli has many therapeutic potentials. Commonly used in aromatherapy, patchouli has been shown to exert antidepressant-like activity by decreasing cortisol and increasing dopamine and serotonin, and it may be a potential contributing mechanism by which positive and negative effects and mood were improved in this study [33]. The effect of aromatherapy on mood also points to another potential advantage of incorporating botanical ingredients in topical formulations. Given that acne is associated with feelings of low self-esteem, embarrassment, and limitations on daily activities of living and social interactions, more research should be conducted to understand how the scent offered by botanicals in topical products may impact the skin–brain axis [33,34].

Of note, this was an open-label study and there was no comparator group. A placebo control is not possible with a regimen as utilized here. While there was no way to assess their individual effects, their various effects have potential to be synergistic. This allows for targeting multiple pathways of a condition simultaneously that may include the skin directly as well as the mind–body connection. However, study results warrant future studies that may utilize a head-to-head design against other regimens/therapies or as an adjuvant to pharmaceutical therapies.

There are several limitations to this study. This study was a pilot study, and the results found here should be investigated with a larger sample size. The study duration was 8 weeks, which allowed us to assess early changes in acne; however, future studies could utilize the results here to justify a longer trial of 12 weeks or longer. Since the products were utilized as a regimen, we can only comment on the effect of the regimen as a whole and not the individual products’ effects. The majority of participants were female. Nevertheless, the efficacy results warrant further study with expansion of the study population to include more males. While this study did not assess the prevalence of depression or anxiety, we were still able to measure statistically significant shifts in mood based on positive and negative effect scores over study duration.

## 5. Conclusions

The use of a botanical skin care regimen (Codex Labs Shaant Balancing regimen) may help to improve the lesion count on the face and trunk in individuals with mild-to-moderate acne, while also improving various aspects of mood. Additional factors such as skin hydration and sebum production may also improve or stabilize during treatment. Further research with larger sample sizes is needed to better understand the benefits and mechanisms of botanical skincare regimens in the prevention and treatment of mild-to-moderate acne.

## Figures and Tables

**Table 1:** Products used.

Products
Shaant Balancing Foam Cleanser
Shaant Balancing Refining Toner
Shaant Balancing Oil Control Cream
Shaant Balancing Exfoliating Facial Scrub
Shaant Balancing Clay Mask
Shaant Balancing Spot Treatment
Shaant Balancing Body Scrub

## Data Availability

No publicly archived datasets.
